# Reduced dietary acid load in U.S. vegetarian adults: Results from the National Health and Nutrition Examination Survey

**DOI:** 10.1002/fsn3.2825

**Published:** 2022-03-15

**Authors:** Maximilian Andreas Storz, Alvaro Luis Ronco

**Affiliations:** ^1^ Centre for Complementary Medicine Department of Internal Medicine II Faculty of Medicine University of Freiburg Freiburg Germany; ^2^ Unit of Oncology and Radiotherapy Pereira Rossell Women's Hospital Montevideo Uruguay; ^3^ School of Medicine CLAEH University Maldonado Uruguay; ^4^ 113076 Biomedical Sciences Center University of Montevideo Montevideo Uruguay

**Keywords:** dietary acid load, epidemiology, NEAP, NHANES, nutrition, plant‐based diet, PRAL, vegetarian

## Abstract

Dietary acid load (DAL) is an important determinant of systemic pH and acid–base homeostasis. Diets abundant in acidogenic foods, such as meat and meat products, induce a low‐grade metabolic acidosis state that has been associated with cardiovascular disease, type‐2‐diabetes, and an increased cancer risk. Fruits and vegetables have alkalizing properties and beneficially affect DAL. It has thus been suggested that a plant‐based diet (restricting or excluding animal products) may be a powerful tool in reducing DAL; yet studies in that particular field are scarce. To explore these associations in greater detail, we examined DAL in self‐identified vegetarians from the United States National Health and Nutrition Examination Survey (2007–2010). We compared dietary intake and two widely used markers of DAL (PRAL (potential renal acid load) and NEAP (net endogenous acid production; NEAP_F_ and NEAP_R_)) among 8,398 nonvegetarians and 191 lacto‐ovo‐vegetarians with reliable dietary intake aged 18 years or older. Vegetarians had a more favorable body mass index and consumed fewer calories (1862.31 kcal/d) than nonvegetarians (2041.12 kcal/d). Vegetarians consumed fewer protein (34.17 g/1000 kcal) and phosphorus compared to nonvegetarians (39.50 g of protein/1000 kcal) but had a higher intake of magnesium and potassium. Nonvegetarians exhibited higher median DAL scores (PRAL: 11.90 mEq/d, NEAP_F_: 53.59 mEq/d, NEAP_R_: 55.67 mEq/d) than vegetarians (PRAL: −0.44 mEq/d, NEAP_F_: 39.60 mEq/d, NEAP_R_: 41.30 mEq/d). Vegetarians had more favorable DAL scores compared to nonvegetarians in this descriptive epidemiologic study. Future (interventional) trials are warranted to examine the varying acid load in different plant‐based dietary patterns.

## Graphical abstract

This study demonstrated that a vegetarian diet is associated with a lower dietary acid load burden. In light of the various health repercussions of a high dietary acid load, our study suggests that plant‐based diets might be a potential strategy to lower systemic acid load.

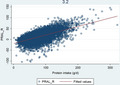

## 1 INTRODUCTION

Acid–base homeostasis is critical to human health and is strongly influenced by the composition of diet (Gannon et al., 2008; Remer, 2000). It is now widely accepted that Dietary Acid Load (DAL) is an important determinant of systemic pH, metabolism, and base–acid equilibrium (Kahleova et al., 2021). A higher DAL has been associated with an increased risk for multiple noncommunicable diseases, including hypertension (Akter et al., 2015), type‐2‐diabetes, and insulin resistance (Williams et al., 2016; Akter et al., 2016; Lee & Shin, 2020, cardiovascular disease (Han et al., 2016), and even certain types of cancer (Ronco et al., 2021a, 2021b).

There is now a general consensus that many plant foods lower DAL (Passey, 2017). Fruits and vegetables produce acid‐neutralizing alkali when metabolized (Osuna‐Padilla et al., 2019; Passey, 2017). In contrast, protein‐rich animal foods often increase DAL. In particular, meat, eggs, and many types of cheese are rich in sulfur‐containing amino acids (cysteine, homocysteine, and methionine) (Adeva & Souto, 2011). These amino acids are catabolized to sulfate (Nakamura et al., 2002; Rehman et al., 2020) thereby increasing DAL. Sulfate excretion is inversely correlated with urinary pH (Adeva & Souto, 2011; Cosgrove & Johnston, 2017). Individuals adhering to plant‐based diets have a lower intake of total protein (Allès et al., 2017), and plant‐based protein has a naturally lower content of sulfur‐containing amino acids (Mariotti & Gardner, 2019).

Foods that are rich in phosphate may also supply acid equivalents to the human diet, depending on the cation that is attached to the phosphate anion (Passey, 2017). A popular example is phosphoric acid (H_3_PO_4_) in dairy products and certain soft drinks (Fernando et al., 1999; Passey, 2017). Protons from dietary H_3_PO_4_ acidify urine, with the excreted form being H_2_PO_4_^−^. The contribution of protons derived from H_3_PO_4_ may be balanced by intestinal absorption of organic anions abundantly found in fruits and vegetables, such as citrate and malate, which upon catabolism undergo conversion to bicarbonate, thus affording alkalinization (Osuna‐Padilla et al., 2019; Scialla & Anderson, 2013).

The difference between these acidic and alkaline products yields the dietary acid load (Cosgrove & Johnston, 2017; Scialla & Anderson, 2013). In light of the accumulating evidence that a high acid load burden from the diet is associated with many adverse health conditions (Engberink et al., 2012; Fagherazzi et al., 2014; Remer & Manz, 1995), dietary strategies to lower DAL are urgently warranted.

Several recent studies highlighted that a plant‐based diet may reduce DAL (Cosgrove & Johnston, 2017; Deriemaeker et al., 2010; Ströhle et al., 2011), however, the number of trials examining this association is limited. The low‐fat vegan diet in particular has been associated with significant reductions in DAL (Kahleova et al.,^2021^) and a 2011 study suggested that vegan diets are more effective than vegetarian diets in lowering DAL scores (Ströhle et al., 2011).

To explore these associations in greater detail, we examined a well‐described sample of self‐identified vegetarians from the United States (U.S.) National Health and Nutrition Examination Survey (NHANES) (Juan et al., 2015). Food intake patterns in this subpopulation have been already analyzed in detail by Juan et al. (2015). The NHANES vegetarian subpopulation between 2007 and 2010 was characterized by a high proportion of lacto‐ovo‐vegetarians and comprised a very small proportion of vegans (Juan et al., 2015). Compared to nonvegetarians in the U.S. population, this group consumed significantly less meat, poultry, solid fats, and added sugars (Juan et al., 2015). Food intake in both groups did not significantly differ with regard to dairy, eggs, fruit, and vegetables, however, the vegetarians consumed more soy foods, legumes, and whole grains.

The aims of the present study were twofold: (a) to investigate potential associations of a vegetarian diet and dietary acid load in an existing cross‐sectional dataset (NHANES), and (b) to contrast the result to other studies that examined the effects of a plant‐based diet on DAL (Cosgrove & Johnston, 2017; Kahleova et al., 2021; Ströhle et al., 2011).

## 2 MATERIALS AND METHODS

### 2.1 Data source and population

#### 2.1.1 Data source

For this project, we used aggregated cross‐sectional data from the Nutrition and Health Examination Surveys (NHANES). The NHANES is one of several health‐related programs conducted by the Centers for Disease Control and Prevention's National Center for Health Statistics in the United States (NHANES, 2020). NHANES is an ongoing project designed to assess the general health and nutritional status of children and adults in the United States (Centers for Medicare & Medicaid Services; NHANES). The NHANES combine (in‐person) interviews, physical examinations, and administer tests of physical activity and fitness.

The NHANES are periodic cross‐sectional surveys that collect data on demographics, diet and other health behaviors (Mazidi et al., 2017, 2018). They were designed to represent the total civilian noninstitutionalized population in the United States and apply a complex multistage probability sampling procedure (Mazidi et al., 2017; Stookey, 2019). This procedure ensures adequate ethnic/racial representation and selection of participants from various geographical regions (Mazidi et al., 2018).

Specifically‐trained interviewers collected demographic, anthropometric, dietary, socio‐economic data, and other information during so‐called home visits. All interviewers completed a comprehensive two‐week training program and many of them already had prior interviewing experience (NHANES, 2010 Questionnaire Data).

The objective of the dietary interview was to estimate total intake of food energy (calories), nutrients, and non‐nutrient food components from foods and beverages that were consumed during a 24‐h period prior to the interview (midnight to midnight) (NHANES, 2010 Dietary Recall). Dietary recall interviews were done on two different days: the first dietary recall interview was collected in‐person in the Mobile Examination Clinic (MEC), whereas the second interview was collected by telephone 3–10 days later (National Health and Nutrition Examination Survey Dietary Interview, 2010). All procedures were carried out in accordance with relevant regulations, ethical protocols, and approved guidelines (Mazidi et al., 2018). All participants provided informed consent before the examination stages and the interviews. A more detailed description of the anthropometric and dietary intake data assessment can be found in a recent paper by Stookey (2019).

Large parts of the NHANES database are publicly available and used by scientists and clinicians worldwide in order to gain deeper insights into nutrition‐related health questions (Dong et al., 2020).

### 2.2 Population

Our analysis is based on the first day of the dietary interview component. We appended data from the 2007–2008 and 2009–2010 surveys to increase the sample size for analyses stratified by population sub‐group (self‐reported vegetarians). A total of 20,686 individuals participated in the NHANES during the aforementioned period, and *n* = 17,359 had a full dataset (no missing values on any variable of interest for our study).

Although the total number of participants was 20,686, our analysis was limited to 8589 individuals based on our predetermined inclusion criteria. These included: age ≥18 years, a reliable dietary status (a NHANES variable indicating the quality and completeness of a survey participant's response to the dietary recall section), plausible self‐reported energy intake data, and available body measures from each participant. Only participants with a minimum intake of 750 kcal/d and a maximum intake of 4000 kcal/d were considered eligible for this analysis. Anthropometric measures were necessary for the various DAL calculations (see below). The assessment of vegetarian status was based on the question “Do you consider yourself to be a vegetarian?”; and was thus based on a (subjective) self‐evaluation.

### 2.3 Dietary acid load calculations

Our methods have been explained elsewhere in detail (Müller et al., 2021). In brief, we employed three commonly used formulas to estimate DAL (Frassetto et al., 1998; Remer & Manz, 1994). These formulas were introduced by Remer and Manz (1994) and Frassetto et al. (1998) and are both frequently used in epidemiological studies and clinical trials.

In a first step, we calculated Potential Renal Acid Load (PRAL) of a diet as follows:

$$\begin{array}{c} \begin{array}{c} \text{PRAL}\;\left(\text{mEq}/\text{day}\right)=\left(0.49\times \text{total protein}\;[g/\text{day}]\right)\\ \;+\left(0.037\;\times \;\text{phosphorus}\;[\text{mg}/\text{day}]\right)\\ \;‐\left(0.021\times \text{potassium}\;[\text{mg}/\text{day}]\right)\\ \;‐\left(0.026\times \text{magnesium}\;\text{[mg}/\text{day]}\right)\\ \;‐\left(0.013\;\times \;\text{calcium}\;\left[\text{mg}/\text{day}\right]\right) \end{array} \end{array}$$

This formula includes intestinal absorption rates for the following nutrients: calcium, magnesium, phosphate, potassium, and protein. Furthermore, it considers ionic dissociation and sulfur metabolism (Remer & Manz, 1994). Remer and Manz (1994) validated this method against urinary renal net acid excretion and found that it reliably predicts the acid load from diet. Positive PRAL values reflect an acid‐forming potential, whereas negative PRAL scores reflect an alkaline‐forming potential (Remer & Manz, 1995).

In a second step, we calculated Net Endogenous Acid Production (NEAP). For this, we used two different formulas: NEAP_F_ (a formula proposed by Frassetto et al. (1998)) and NEAP_R_ (a formula proposed by Remer and Manz (1994) and Remer et al., 2003).

The formula by Remer et al. estimates net endogenous acid production from average intestinal absorption rates of ingested protein and micronutrients (as reflected in the PRAL‐score) and also considers anthropometry‐based estimates for organic acid excretion (OA_est_):

$${\text{Estimated NEAP}}_{R}\left(\text{mEq}/d\right)=\text{PRAL}\left(\text{mEq}/d\right)+{\text{OA}}_{\text{est}}\left(\text{mEq}/d\right)$$

We calculated OA_est_ (mEq/d) as follows:

Individual body surface area×41/1.73

In order to calculate body surface area, we used the formula of Du Bois and Du Bois: Body surface area (m^2^) = [0.007184 × height (cm)^0.725^ × weight(kg)^0.425^].

Finally, we also calculated net endogenous acid production based on a formula by Frassetto et al. (NEAP_F_) (2007). This formula considers the potassium and protein content of diet:

Estimated NEAP_F_ (mEq/d) = [54.4 x protein (g/d) / potassium (mEq/d)] −10.2

Both algorithms have their merits and drawbacks (Ströhle et al., 2011); thus we employed both models (NEAP_F_ and NEAP_R_) and examined their associations with a vegetarian diet.

### 2.4 Statistical analysis

We used STATA 14 statistical software (StataCorp. 2015. Stata Statistical Software: Release 14. College Station, TX: StataCorp LP) for our analysis. We used both the “. svyset” and “. svy” commands to account for the complex NHANES survey design characteristics and the population weights.

We included the primary sampling unit variable for variance estimation (and the pseudo‐stratum variable as the stratification variable) that were provided in the NHANES datasets. The variable “sdmvstra” (for the masked variance unit pseudo‐stratum) and the variable “sdmvpsu” (for the masked variance unit) were used. Additional data on sampling design of the NHANES and both variables may be obtained from the NHANES “sample design module” (National Health and Nutrition Examination Survey Tutorials, 2021). Both datasets included a day 1 dietary intake weight that must be used when working with dietary data from day 1. Since we appended two different datasets (2007–2008 and 2009–2010), we generated a 4‐year weight for dietary data (wtdrd4y = wtdrd1/2).

A previous analysis by Juan and colleagues (2015) examined food intake patterns in vegetarians from those surveys and revealed that this group had a significantly lower total calorie intake (compared to nonvegetarians). To account for this phenomenon, and to evaluate micro‐ and macronutrient intake in relation to total energy intake, we employed a commonly used energy adjustment method (National Cancer Institute, 2020 ‐ Dietary Assessment Primer). In this study, nutrient density is expressed as intake (in gram or milligram)/1000 kcal.

We used histograms, box plots, and subpopulation summary statistics to check for frequency distribution and normality of the data before starting our analysis. We described normally distributed variables with mean ± standard deviation and non‐normal distributed variables with median (interquartile range). We used student's *t*‐tests to compare intergroup differences in macro‐ and micronutrients and DAL scores if the variable was normally distributed (and did not include significant outliers), otherwise, we adopted the Mann–Whitney U (Wilcoxon rank sum) test. For categorical variables, we used STATA’s design‐based Rao–Scott *F*‐test to test for potential associations. Finally, we used Sribney's (Sribney, 2014, STATACorp) manual to estimate correlations and their level of significance with survey data.

## 3 RESULTS

**FIGURE 1:**
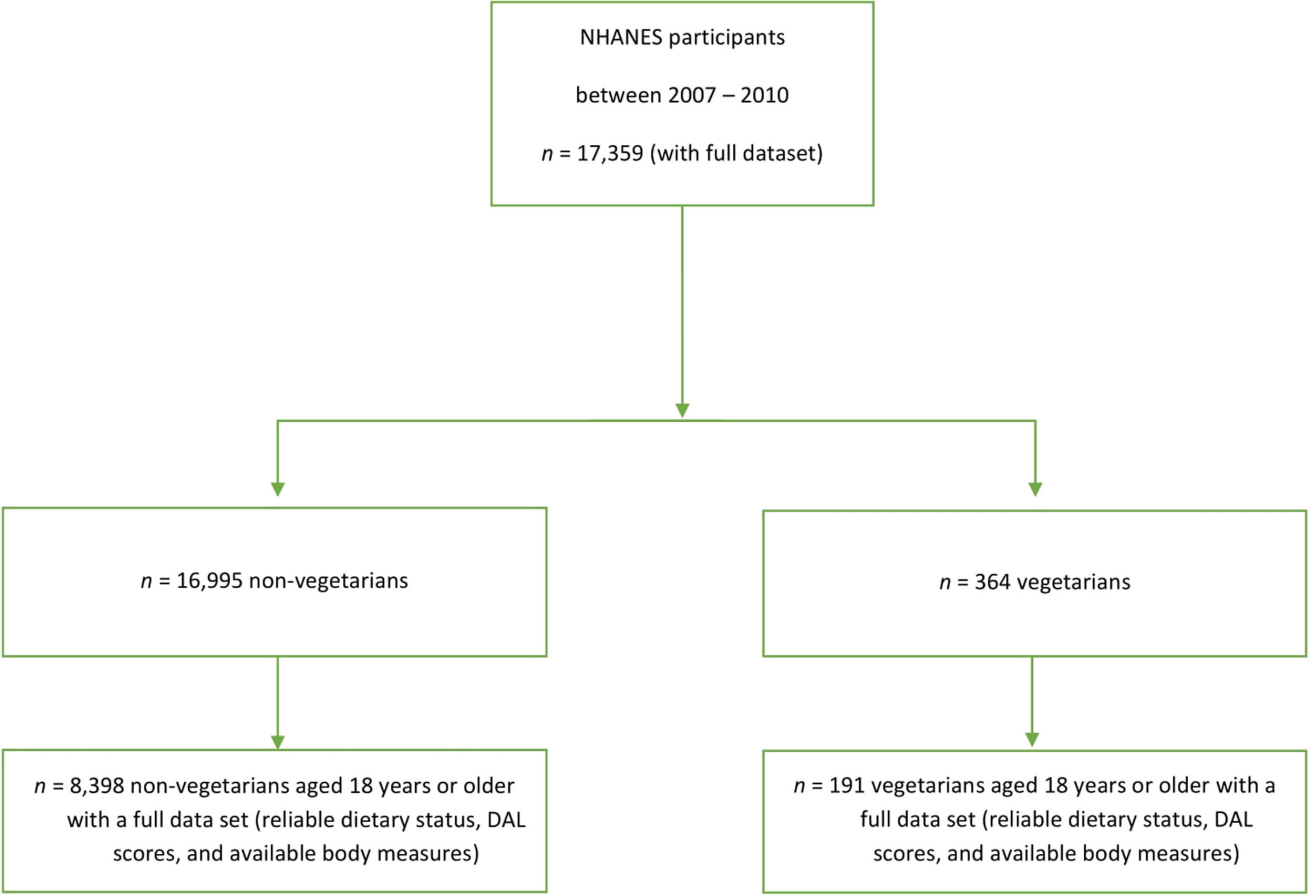
Patient inclusion flow diagram

A total of *n* = 8589 individuals from NHANES 2007–2010 were included in our analysis. The sample included *n* = 191 (self‐perceived) vegetarians and *n* = 8398 nonvegetarians aged 18 years or older. Figure 1 shows a participant inclusion flow chart for our study. We excluded *n* = 113 vegetarians for being younger than 18 years; *n* = 42 were excluded for unavailable anthropometric data, and *n* = 18 were excluded for implausible self‐reported energy intake data. From the general population, *n* = 5649 participants for being <18 years, *n* = 2204 participants for a lack of available anthropometric data, and *n* = 744 participants for implausible self‐reported energy intake data.

All subjects had a reliable dietary status. Table 1 shows anthropometric and demographic data of the participants in this particular sample.

**TABLE 1:** Demographic and anthropometric data of our sample

	Self‐perceived vegetarians (*n* = 191)	Nonvegetarians (*n* = 8398)	*p*‐value
Age (years)	42.90 ± 17.69	45.96 ± 17.45	*p* = .051
Sex			
Males	33.01%	47.60%^a^	*p* = .021
Females	66.99%	52.40%^a^	
Weight (kg)	67.26 ± 13.66	77.69 ± 17.57	*p* < .001
Height (cm)	165.41 ± 9.94	168.84 ± 9.94	*p* = .004
Body Mass Index (kg/m²)	24.52 ± 4.24	27.16 ± 5.26	*p* < .001

Note

Gender distribution is based on weighted proportions. Normally distributed data are shown as mean ± SD; not normally distributed data are shown as medians (interquartile range). P‐values are based on student's *t*‐tests to compare intergroup differences. The *p*‐value for sex is based on STATA's design‐based Rao–Scott *F*‐test and tests for a potential association between vegetarian status and gender.

^a^ Indicates significant differences in the weighted proportions.

Self‐perceived vegetarians had a significantly lower body weight and body mass index than nonvegetarians (*p *= <.05). Approximately 2/3 of the self‐perceived vegetarian group were female, whereas the gender distribution among nonvegetarians was more equal (Table 1).

Table 2 shows the total calorie intake (in kcal/day) and the energy‐adjusted daily intake of selected micro‐ and macronutrients in both groups (either in gram/1000 kcal or mg/1000 kcal).

**TABLE 2:** Macro‐ and micronutrient intake among self‐identified vegetarians and nonvegetarians

	Self‐perceived vegetarians (*n* = 191)	Nonvegetarians (*n* = 8398)	*p*‐value
Calories (kcal)/day	1862.31 ± 740.96	2041.12 ± 733.12	*p* = .020
Protein (gram/1000 kcal)	34.17 ± 9.02	39.50 ± 11.90	*p *= <0.001
Potassium (mg/1000 kcal)	1434.92 ± 472.14	1342.73 ± 472.31	*p* = .195
Magnesium (mg/1000 kcal)	172.33 ± 61.59	148.36 ± 53.53	*p* = .003
Phosphorus (mg/1000 kcal)	641.75 ± 145.08	659.25 ± 184.45	*p* = .205
Calcium (mg/1000 kcal)	502.37 ± 210.35	473.22 ± 231.09	*p* = .257

Note

Normally distributed data are shown as mean ± SD; not normally distributed data are shown as medians (interquartile range).

Self‐perceived vegetarians consumed significantly fewer calories than nonvegetarians (see Table 2). Nonvegetarians consumed significantly more protein (39.50 g/1000 kcal) compared to vegetarians (34.17 g/1000 kcal), whereas self‐perceived vegetarians had a significantly higher magnesium intake (see Table 2). Vegetarians also had a higher intake of potassium and calcium; however, the intergroup difference was not statistically significant.

Table 3 shows the different DAL scores for both groups. Histograms suggested a normal distribution for all DAL scores, whereas box‐plots revealed a few outliers in both groups. Thus, we decided to use the nonparametric Mann–Whitney U (Wilcoxon rank sum) test to examine intergroup differences instead of the parametric student's *t*‐test.

**TABLE 3:** Dietary acid load scores in self‐identified vegetarians and nonvegetarians: a comparison

	Self‐perceived vegetarians (*n* = 191)	Nonvegetarians (*n* = 8398)	*p*‐value
PRAL (mEq/d)	−0.44 (−12.19 to 11.01)	11.90 (−1.15 to 25.66)	*p *= <.001
NEAP_R_ (mEq/d)	41.30 (28.63 to 52.49)	55.67 (42.07 to 70.95)	*p *= <.001
NEAP_F_ (mEq/d)	39.60 (31.48 to 52.07)	53.59 (40.28 to 70.48)	*p *= <.001

Note

All DAL indexes were not normally distributed, hence data are shown as median (interquartile range).

All three DAL scores (PRAL and both NEAP scores based on the Frasetto and Remer formula) were lower in the vegetarian group than in the nonvegetarian group.

**FIGURE 2:**
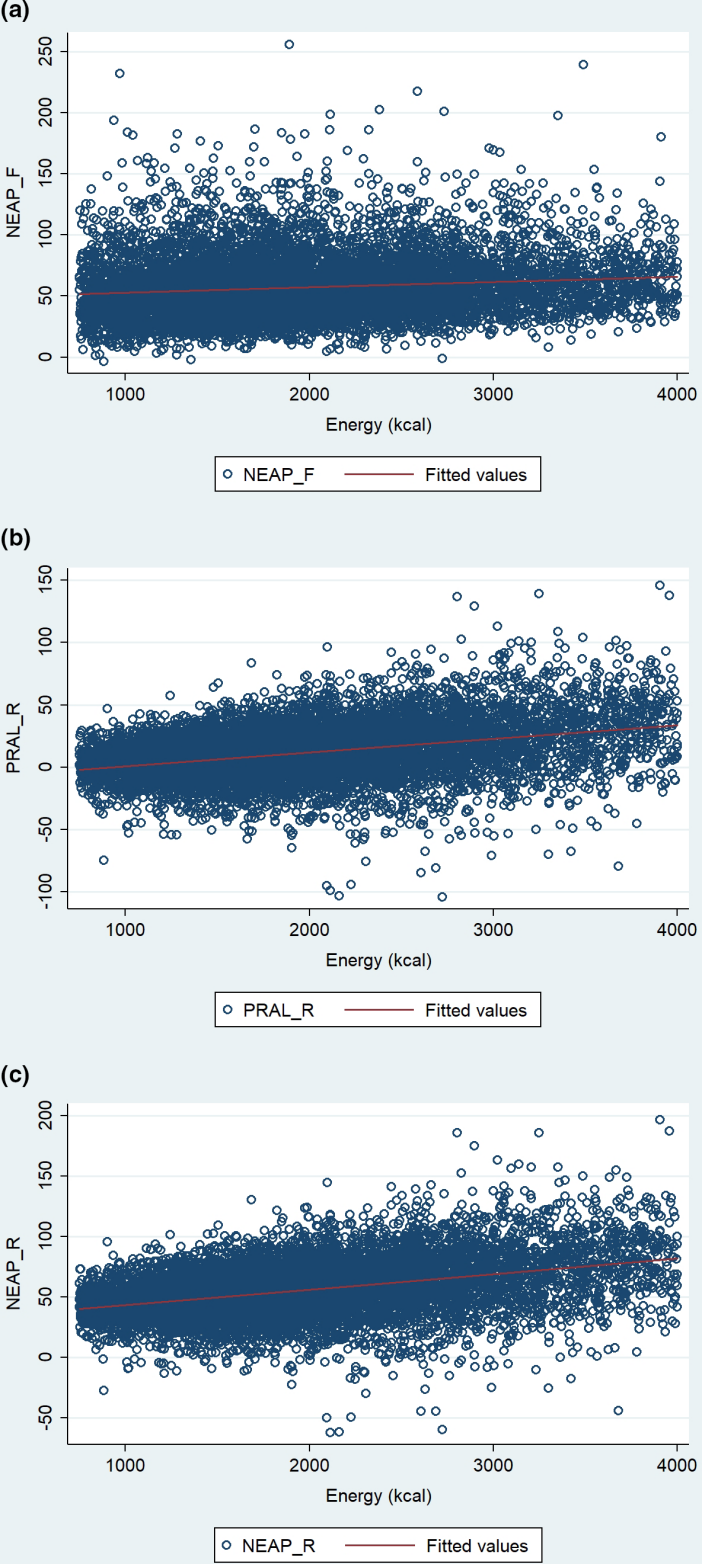
Weighted scatterplots of total calorie intake and all 3 DAL scores (entire sample); including (a) NEAP_F_; (b) PRAL_R_; and (c) NEAP_R_

A Pearson's product‐moment correlation was run to assess the relationship between DAL scores and total calorie intake in all participants (*n* = 8589) in the sample (see Figure 2). There was a moderate positive correlation between total calorie intake and the NEAP_R_ score (see Figure 2c), r = 0.40, *p*<.0001. Correlation coefficient value for PRAL_R_ and total calorie intake was r = 0.36 (Figure 2b; *p* <.001). The strength of association was weaker for the NEAP_F_ score and total calorie intake (Figure 2a; r = 0.12 with *p* <.001).

**FIGURE 3:**
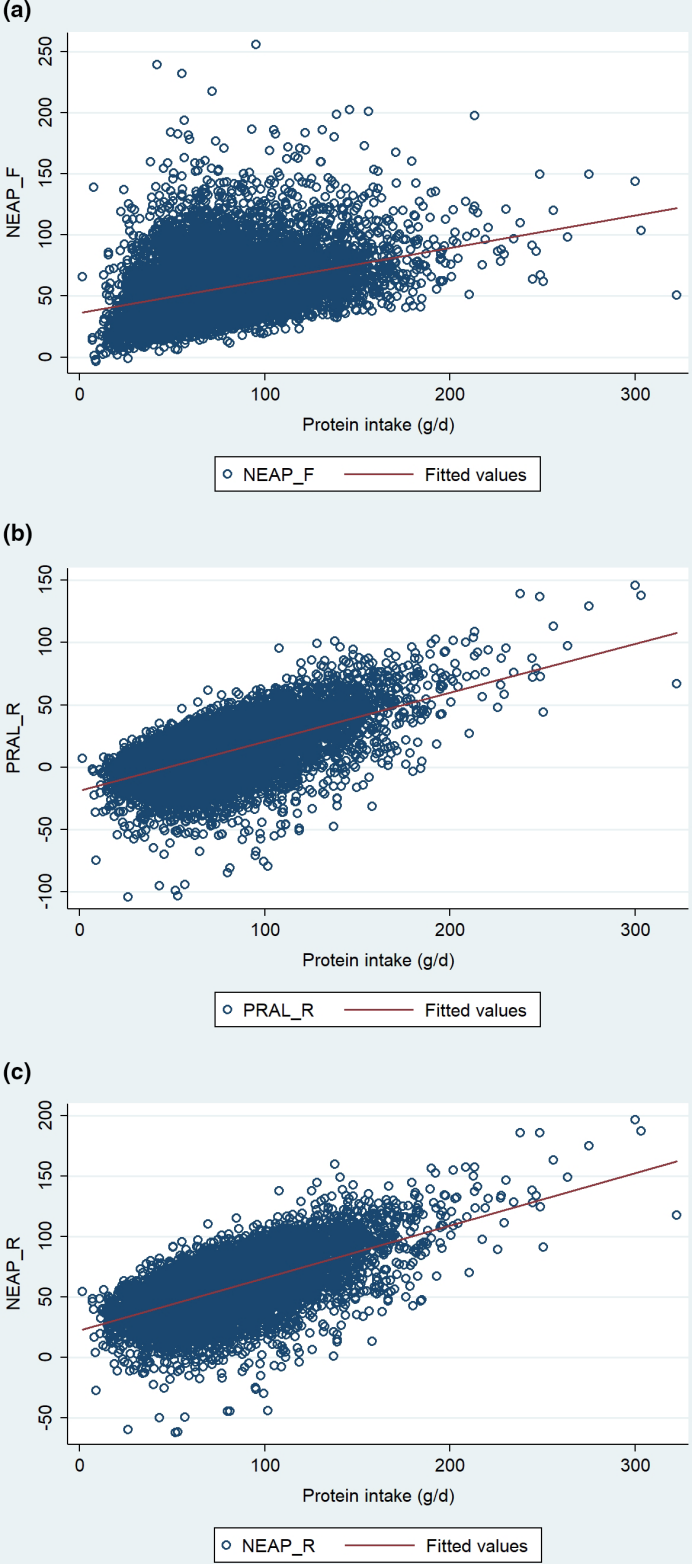
Weighted scatterplots of total protein intake (g/d) and all 3 DAL scores (entire sample); including (a) NEAP_F_; (b) PRAL_R_; and (c) NEAP_R_

Finally, we also used a Pearson's product‐moment correlation to assess the relationship between DAL scores and total protein intake in all participants (*n* = 8,589) in the sample (see Figure 3).

We found a strong positive correlation between total protein intake and the PRAL_R_ score (see Figure 3b): r = 0.61, *p* <.001. A comparable association was found for the NEAP_R_ score (Figure 3c): r = 0.63, *p* <.001. Correlation coefficient value for NEAP_F_ and total protein intake was r = 0.37 (Figure 3a; *p* <.001).

## 4 DISCUSSION

This descriptive epidemiologic study sought to examine different DAL scores in self‐perceived vegetarians and nonvegetarians in a large cross‐sectional NHANES sample (2007–2010). Our study included 8589 individuals of which *n* = 191 were self‐perceived vegetarians. DAL scores were significantly lower in this group (as compared to nonvegetarians). Vegetarians consumed fewer protein and phosphorus compared to nonvegetarians but had a higher intake of magnesium and potassium.

Our study adds to the growing body of evidence that a plant‐based diet is associated with a more favorable DAL profile (Cosgrove & Johnston, 2017; Deriemaeker et al., 2010; Kahleova et al., 2021; Müller et al., 2021; Ströhle et al., 2011). Based on our analysis, self‐perceived vegetarians had a median PRAL score of −0.44 mEq/d, indicating a slight alkaline‐forming potential (Cosgrove & Johnston, 2017), whereas nonvegetarians had a positive median PRAL score (11.90 mEq/d), indicating an acid forming potential. Median NEAP scores were also lower in the vegetarian group (39.60 and 41.30 mEq/d), whereas nonvegetarian had a median NEAP value ≥53 mEq/d for both scores. Thus, we believe that our findings are consistent with the literature as discussed below.

Lemann (1999) estimated that the average diet consumed by (apparently healthy) omnivorous U.S. adults generates approximately 50 mEq of acid per day. Gannon et al. (2008) reported a median NEAP value of 51 mEq/d in men aged 65–71. Our results in the nonvegetarian group are somewhat similar (53.59 mEq/d for NEAP_F_ and 55.67 mEq/d for NEAP_R_). In contrast, those individuals consuming a plant‐based diet usually have lower PRAL and NEAP scores. Vegetarians in a Belgian study had a mean PRAL value of −5.4 (±14.4) mEq/d, whereas their omnivorous counterparts had a mean PRAL value of 10.3 (±14.4; Deriemaeker et al., 2010).

Glancing at interventional trials that investigated the acid load‐lowering effects of a vegan diet (Cosgrove & Johnston, 2017; Kahleova et al., 2021; Müller et al., 2021), a vegetarian dietary pattern seems to be less effective than a (strict) vegan diet. Kahleova and colleagues (2021) recently examined the effects of an ad libitum low‐fat vegan diet on DAL. After just 16 weeks, median PRAL values fell from 3.6 (0.4 to 6.8) mEq/d to −20.7 (−23.3 to −18.1) mEq/d. In the same cohort, median NEAP values fell from 50.8 (47.1 to 54.5) mEq/d to 25.7 (24.0 to 27.4). Cosgrove and Johnston (2017) reported comparable findings: after seven consecutive days on a (strict) vegan diet, mean PRAL values fell from 23.7 ± 17.7 to −6.0 ± 12.8.

One reason to explain the lower DAL scores in vegans (as opposed to lacto‐ovo‐vegetarians) is that their diets usually exclude all animal foods; that is not only meat, poultry, and fish but also dairy, cheese, and eggs (Storz, 2020). These foods often contain large amounts of dietary phosphorus and preservative phosphate (phosphoric acid, polyphosphates) (D’Alessandro et al., 2015; Kahleova et al., 2021), which have a high gastrointestinal absorption rate and thereby contribute to an elevated DAL (D’Alessandro et al., 2015). Gannon et al. (2008) reported a significantly positive association between acid load and protein intake, phosphorus intake and total energy intake. The nonvegetarians in our sample exhibited a higher intake of both of these nutrients (see Table 2), a factor that certainly contributed to their DAL.

Our findings align well with previous studies in the field of plant‐based nutrition and DAL (Cosgrove & Johnston, 2017; Deriemaeker et al., 2010; Kahleova et al., 2021). A plant‐based diet has the potential to lower DAL, yet the composition of diet appears to play a crucial role. Some people add large amounts of dairy products to their plant‐based diet—foods that are often high in phosphoric acid (H_3_PO_4_) which is an important contributor to DAL. Plant foods also contain phosphorus, yet in the form of phytate, which has a lower bioavailability and, therefore, no acidifying effects (Osuna‐Padilla et al., 2019; Kahleova et al., 2021).

In light of the numerous health repercussions of a high DAL (Osuna‐Padilla et al., 2019), a well‐designed nutrient‐dense plant‐based diet could be a potential solution. Additional randomized‐controlled trials could help to differentiate the acid‐lowering effects of different plant‐based diets (lacto‐ovo‐vegetarian, vegan). As there are currently no reference values for PRAL and NEAP scores, such trials could be helpful to gain a deeper understanding of “common” DAL scores in the respective populations.

Finally, there is an urgent need for interventional studies that investigate the effects of DAL‐lowering diets in relation to specific diseases. A high dietary acid burden has been associated with numerous adverse health conditions, including type‐2‐diabetes and insulin resistance (Akter et al., 2016; Lee & Shin, 2020; Williams et al., 2016). As of recently, studies began to evaluate the impact of DAL‐lowering diets on specific disease‐related endpoints, such as body weight and insulin sensitivity in type‐2‐diabetes (Kahleova et al., 2021). Additional clinical (interventional) trials that comparably analyze the effects of a DAL reduction are urgently warranted with regard to many other chronic (noncommunicable) diseases.

### 4.1 Strengths and limitations

The present analysis has several strengths and limitations that warrant further discussion. One of the main strengths is the large dataset that is based on a nationally representative population‐based survey. We included a relatively high number of vegetarians (*n* = 191) with reliable dietary data and a large “control group”. Furthermore, our analysis includes two different NEAP scores, as opposed to many studies that estimated NEAP solely on the Frassetto formula. Our findings align well with the existing literature on DAL in individuals consuming a plant‐based diet and may help to establish adequate reference values for this group.

However, our study also has several limitations. The (descriptive) epidemiologic nature of our study warrants caution when comparing the results of the present analysis with other (interventional) trials. Our findings rely on observational data, which is deemed to be inferior to experimental data in determining causality (Satija et al., 2015). Another weakness of our study is that the “vegetarian status” of participants was captured with the question “Do you consider yourself to be a vegetarian?” during the MEC interview. This leaves a lot of room for interpretation, and, in fact, some self‐identified vegetarians reported eating meat or fish over a 24‐h period according to Juan et al. (2015). Thus, the examined subpopulation also includes several semi‐vegetarians. As meat products are usually acidogenic (Kahleova et al., 2021), we might have underestimated the “true” DAL‐lowering effect of the lacto‐ovo‐vegetarian diet consumed by most individuals in our analysis. It is also important to note that cross‐sectional designs can result in biases, such as recall‐bias during the dietary interview. An additional limitation is that our analysis only includes dietary data from day 1, because adding data from the second day would have further reduced the total sample size (and particularly the number of vegetarians with a complete dataset). On the other hand, using data from day 1 only is a widely employed and accepted strategy that has been considered reliable to assess dietary intake (Stookey, 2019).

Another point worth mentioning is that our analysis did not account for supplement usage (e.g., calcium supplements for osteoporosis prevention), which could be considered a potential confounder. Finally, the time of data acquisition might play an important role. The data used in this analysis stem from the years 2007 to 2010. Recent publications using data from food composition tables indicated a downward trend in the mineral and trace element content of many plant foods, probably as a consequence of intensive farming practices (resulting in soil depletion). It is not inconceivable that this might indirectly affect the DAL‐lowering effects of fruits and vegetables, however, this potential confounder is beyond the scope of an epidemiological paper (Ekholm et al., 2007; Fan et al., 2008).

## 5 CONCLUSION

Results from our study demonstrate that plant‐based diets are associated with a lower acid load burden. In light of the various health repercussions of a high DAL and the recently published studies by Kahleova et al. (2021) and Cosgrove and Johnston (2017), it is conceivable that a plant‐based diet might be a potential strategy to lower systemic acid load. Additional studies are required to gain deeper insights into the varying acid load‐lowering effects of the different plant‐based dietary patterns.

## CONFLICT OF INTEREST

The authors declare no conflict of interest.

## AUTHOR CONTRIBUTIONS

**Maximilian Andreas Storz:** Conceptualization (lead); Data curation (lead); Formal analysis (lead); Methodology (lead); Project administration (lead); Visualization (equal); Writing – original draft (lead). **Alvaro Luis Ronco :** Formal analysis (equal); Methodology (equal); Project administration (equal); Validation (equal); Visualization (supporting); Writing – original draft (supporting); Writing – review & editing (supporting).

## Data Availability

The specific dataset associated with this study will be made available by the corresponding author upon reasonable request.
